# A voxel‐based asymmetry study of the relationship between hemispheric asymmetry and language dominance in Wada tested patients

**DOI:** 10.1002/hbm.24058

**Published:** 2018-03-23

**Authors:** Simon S. Keller, Neil Roberts, Gus Baker, Vanessa Sluming, Enis Cezayirli, Andrew Mayes, Paul Eldridge, Anthony G. Marson, Udo C. Wieshmann

**Affiliations:** ^1^ Department of Molecular and Clinical Pharmacology Institute of Translational Medicine, University of Liverpool Liverpool United Kingdom; ^2^ Department of Psychological Sciences Institute of Psychology, Health and Society, University of Liverpool Liverpool United Kingdom; ^3^ The Walton Centre NHS Foundation Trust Liverpool United Kingdom; ^4^ Edinburgh Imaging, The Queens Medical Research Institute (QMRI), School of Clinical Sciences University of Edinburgh Edinburgh United Kingdom; ^5^ School of Medicine University of St Andrews Scotland United Kingdom; ^6^ School of Psychological Sciences University of Manchester Manchester United Kingdom

**Keywords:** Broca's area, cerebral asymmetry, epilepsy, insula, language lateralization

## Abstract

Determining the anatomical basis of hemispheric language dominance (HLD) remains an important scientific endeavor. The Wada test remains the gold standard test for HLD and provides a unique opportunity to determine the relationship between HLD and hemispheric structural asymmetries on MRI. In this study, we applied a whole‐brain voxel‐based asymmetry (VBA) approach to determine the relationship between interhemispheric structural asymmetries and HLD in a large consecutive sample of Wada tested patients. Of 135 patients, 114 (84.4%) had left HLD, 10 (7.4%) right HLD, and 11 (8.2%) bilateral language representation. Fifty‐four controls were also studied. Right‐handed controls and right‐handed patients with left HLD had comparable structural brain asymmetries in cortical, subcortical, and cerebellar regions that have previously been documented in healthy people. However, these patients and controls differed in structural asymmetry of the mesial temporal lobe and a circumscribed region in the superior temporal gyrus, suggesting that only asymmetries of these regions were due to brain alterations caused by epilepsy. Additional comparisons between patients with left and right HLD, matched for type and location of epilepsy, revealed that structural asymmetries of insula, pars triangularis, inferior temporal gyrus, orbitofrontal cortex, ventral temporo‐occipital cortex, mesial somatosensory cortex, and mesial cerebellum were significantly associated with the side of HLD. Patients with right HLD and bilateral language representation were significantly less right‐handed. These results suggest that structural asymmetries of an insular‐fronto‐temporal network may be related to HLD.

## 1 INTRODUCTION

Interhemispheric structural asymmetries of cortical regions known to play an important role in language function have been considered as candidate regions representing an anatomical basis for hemispheric language dominance (HLD), that is, the cerebral hemisphere most dominant for expressive and receptive language functions (Keller, Crow, Foundas, Amunts, & Roberts, 2009a; Toga & Thompson, 2003; Witelson & Kigar, 1988). The left cerebral hemisphere is dominant for language in up to 90% of right‐handed people (Knecht et al., 2000a, 2000b). This figure decreases in people with left‐handedness (Knecht et al., 2000b; Pujol, Deus, Losilla, & Capdevila, 1999) and left‐sided brain insult (Brazdil, Zakopcan, Kuba, Fanfrdlova, & Rektor, 2003; Liegeois et al., 2004; Pahs et al., 2013). There is a wealth of evidence indicating that structural differences exist between the two hemispheres. These regions include posterior language regions such as planum temporale and posterior perisylvian cortex (Dorsaint‐Pierre et al., 2006; Foundas, Leonard, Gilmore, Fennell, & Heilman, 1994; Galaburda, LeMay, Kemper, & Geschwind, 1978a; Galaburda, Sanides, & Geschwind, 1978b; Geschwind, 1972, 1978; Geschwind & Levitsky, 1968; Josse, Mazoyer, Crivello, & Tzourio‐Mazoyer, 2003; Josse & Tzourio‐Mazoyer, 2004; Tzourio, Nkanga‐Ngila, & Mazoyer, 1998), anterior speech regions including Broca's area (Amunts, Schleicher, Ditterich, & Zilles, 2003; Amunts & Zilles, 2006; Falzi, Perrone, & Vignolo, 1982; Foundas, Leonard, Gilmore, Fennell, & Heilman, 1996; Keller et al., 2009a, 2007, 2011; Keller, Roberts, & Hopkins, 2009b; Knaus, Corey, Bollich, Lemen, & Foundas, 2007; Tomaiuolo et al., 1999; Uylings, Jacobsen, Zilles, & Amunts, 2006; Wada, Clarke, & Hamm, 1975), insula (Biduła & Króliczak, 2015; Chiarello, Vazquez, Felton, & Leonard, 2013; Greve et al., 2013; Keller et al., 2011), and interconnecting white matter tracts (Barrick, Lawes, Mackay, & Clark, 2007; Catani, Jones, & Ffytche, 2005; Nucifora, Verma, Melhem, Gur, & Gur, 2005; Powell et al., 2006; Sreedharan, Menon, James, Kesavadas, & Thomas, 2015; Vernooij et al., 2007). However, whether or not structural asymmetries underlie HLD is unresolved (Keller et al., 2009a; Witelson & Kigar, 1988).

Presurgical evaluation of patients with epilepsy has provided the opportunity to assess the relationship between HLD and structural asymmetries through information provided by the Wada test and magnetic resonance imaging (MRI), respectively. The Wada test offers the most unequivocal measure of HLD, and is the gold standard against which imaging measures of HLD, including functional MRI, magnetoencephalography, and functional transcranial Doppler sonography, have been compared (Abou‐Khalil, 2007; Baxendale, 2009; Binder et al., 1996; Desmond et al., 1995; Kemp et al., 2018; Knecht et al., 1998). The invasive nature of Wada testing makes the application constrained to presurgical evaluation of patient cohorts. However, studies that have investigated relationships between cerebral asymmetries and HLD in Wada tested patients offer the most reliable lateralization of hemispheric language functions, whereas reproducibility issues may arise from functional neuroimaging of HLD (Ruff et al., 2008; Rutten, Ramsey, van Rijen, & van Veelen, 2002).

So far, only a few studies have investigated the relationship between cerebral asymmetries and HLD in Wada tested patients. Using manual delineation techniques on MR images, Foundas et al. (1994) reported that 11 patients with language lateralized to the left hemisphere had leftward length asymmetry of the planum temporale, whereas a single patient with right language dominance had a corresponding rightward asymmetry of the same structure. In patients with left HLD, Oh and Koh (2009) reported leftward volume asymmetry of the planum temporale in 30/31 (97%) of patients with right temporal lobe epilepsy (TLE), and in 15/17 (88%) of patients with left TLE. Conversely, 7/8 (88%) patients with left TLE and right HLD had rightward volume asymmetry of the planum temporale, suggesting that directional asymmetry of the planum temporale may be consistent with the laterality of HLD. On the other hand, Dorsaint‐Pierre et al. (2006) reported that manual volume MRI measurements of the planum temporale and adjacent Heschl's gyrus were generally leftward asymmetric, regardless of the side of HLD in 42 Wada tested patients. However, when using voxel‐based MRI methods, the authors reported that left and right HLD patients had a grey matter density bias to the ipsilateral pars opercularis of the inferior frontal gyrus—one of the main structural components of Broca's area (Keller et al., 2009a)—suggesting that structural asymmetry of anterior speech regions may underlie functional lateralization in patients with epilepsy (Dorsaint‐Pierre et al., 2006). Foundas et al. (1996) had similarly earlier reported a relationship between surface area of the pars triangularis of the inferior frontal gyrus and HLD. In the largest sample of Wada tested patients that has been investigated to assess the relationship between HLD and structural asymmetry to date, Charles et al. (1994) reported that the length of the occipital lobe was related to language dominance in 57 patients, an effect that was not related to handedness. These findings were later confirmed in surface area measurements of the occipital pole in 55 patients (Charles et al., 1997). More recent studies using diffusion tensor imaging (DTI) in small groups of Wada tested patients have suggested an asymmetrical structural bias (in density, volume and number of tracts) of the arcuate fasciculus—a white matter bundle connecting anterior (Broca's) and posterior (Wernicke's) speech regions—ipsilateral to the side of HLD (Ellmore et al., 2010; Matsumoto et al., 2008; Sreedharan et al., 2015). A shortcoming of previous imaging studies in Wada tested patients, however, is the use of relatively small heterogeneous patient cohorts, with no control over location of seizure onset, which may influence structural brain asymmetries. Additionally, it is difficult to identify reproducible relationships between HLD and anatomical asymmetry due to interstudy differences in MRI data analysis techniques.

Quantitative assessment of brain structure and HLD in healthy people, as determined using functional neuroimaging techniques (e.g., functional MRI, transcranial Doppler sonography), has provided variable support for some of the findings referred to above in Wada tested patients. In a detailed review, Keller et al. (2009a) report that structural asymmetry of anterior speech regions is not consistently related to HLD. Some studies report leftward structural asymmetry of Broca's area in cohorts of healthy people with presumed left HLD (Toga & Thompson, 2003), whereas others do not (Keller et al., 2007, 2009b). Furthermore, Region‐of‐Interest (ROI) measures of volume, thickness, and surface area asymmetries of the constituent regions of Broca's area have been reported not to be related to the side of HLD in some studies (Greve et al., 2013; Keller et al., 2011). However, in a novel study that correlated voxel‐wise functional MRI BOLD activation with voxel‐wise grey matter density, a positive correlation was reported between extent of leftward language lateralization and grey matter density of Broca's area in the left hemisphere (Josse, Kherif, Flandin, Seghier, & Price, 2009). As reported in studies of Wada tested patients (Dorsaint‐Pierre et al., 2006), leftward interhemispheric asymmetry of the planum temporale exists regardless of the laterality or extent of HLD (Josse et al., 2003; Keller et al., 2011). However, increased volume of left planum temporale was related to an increased leftward lateralization of language functions in perisylvian regions in one study (Josse et al., 2003). The relationship between anatomical asymmetry of insula and HLD appears to be considerably more robust across studies. Keller et al. (2011) first reported that the volume of the insula was leftward asymmetrical in people with left HLD, and there was a rightward asymmetry in people with right HLD. Subsequent studies have confirmed a relationship between insular interhemispheric asymmetry and HLD (Bidula & Kroliczak, 2015; Chiarello et al., 2013; Greve et al., 2013). Some DTI studies have also suggested a relationship between asymmetric features of the arcuate fasciculus and HLD in healthy people (James et al., 2015; Powell et al., 2006), while others have reported consistent leftward structural asymmetry of the arcuate fasciculus regardless of the side of HLD (Vernooij et al., 2007).

The goal of this study was to investigate the relationship between HLD and interhemispheric structural asymmetries in a large sample of consecutive patients with refractory epilepsy in whom Wada test and MRI was performed at the Walton Centre NHS Foundation Trust in Liverpool. We employed a voxel‐based asymmetry (VBA) approach that determines interhemispheric asymmetries of homologous left–right hemispheric voxels over the entire brain with no a priori region of interest. This approach has recently been described in detail (Kurth, Gaser, & Luders, 2015), and earlier versions were applied to determine interhemispheric asymmetries in samples with no known HLD (Good et al., 2001; Luders, Gaser, Jancke, & Schlaug, 2004; Watkins et al., 2001). We first determined whole‐brain interhemispheric asymmetries in patients with left HLD, who represent the majority of the population, and compare asymmetries in these patients with those seen in healthy right‐handed controls to identify whether any asymmetries are attributable to pathologic processes (i.e., structural alterations related to epilepsy). We subsequently directly compared structural asymmetries between patients with left HLD with those with right and bilateral language representation to determine the neuroanatomical correlates of language dominance.

## 2 METHODS

### 2.1 Participants

A total of 135 consecutive patients with medically intractable focal epilepsy who underwent Wada testing at the Walton Centre NHS Foundation Trust in Liverpool in context of presurgical evaluation between 1995 and 2001 were included in this study. All patients were recruited on the basis of having corresponding Wada and presurgical MRI data acquired using the same scanner. Patients with complete clinical histories from a wider population of patients who did not undergo Wada testing or who had incomplete demographic, clinical, or imaging data were excluded. Of the 135 patients, 123 (91%) had presumed TLE (69 left, 46 right, two bilateral, six undetermined laterality) and 12 (9%) had an extratemporal focus (six left, three right, three undetermined laterality). All patients underwent handedness assessment using the Edinburgh Handedness Inventory (EHI) (Oldfield, 1971). For the whole group of patients, mean age was 33.3 years (*SD* 9.68), mean age of onset of epilepsy was 11.1 years (*SD* 8.54), mean duration of epilepsy was 22.2 years (*SD* 10.89), mean handedness score was 75.8 (*SD* 55.55), and included 68 (50.4%) females and 67 males (49.6%). We additionally studied a cohort of 54 neurologically and psychiatrically healthy right‐handed controls who were scanned using the same protocol on the same MRI scanner (mean age 39.1 years, *SD* 12.0; 30 females (55.6%); EHI mean 88.3, *SD* 18.6).

### 2.2 Wada procedure

Each patient underwent the Wada test using a standardized protocol (Kemp, Wilkinson, Caswell, Reynders, & Baker, 2008). Patients received an angiogram, and EEG monitoring was performed throughout the procedure using the International 10–20 system. Access to blood vessels was gained through the femoral artery using the Seldinger technique. Five hundred milligrams of sodium amytal was dissolved in 5 ml saline; 2.5 ml (250 mg) was subsequently drawn up in a 20 ml syringe and the syringe was filled with 17.5 ml saline. The same procedure was performed using a second syringe; the first syringe was used for one hemisphere and the second for the other. Between 100 and 150 mg of sodium amytal was typically required per hemisphere. With the catheter in the carotid artery, sodium amytal was slowly titrated. The carotid artery of the intended side of surgery was injected first, during which time each patient was asked to hold their hands above their head with the arms extended. Neuropsychological testing commenced when the arm contralateral to the injected carotid artery sunk down and the EEG showed slow rhythms over the injected hemisphere. Slowing on the contralateral hemisphere and/or bilateral arm weakness would indicate cross flow of sodium amytal, which renders the test invalid; this did not occur in the 135 patients recruited into this study.

After the necessary dose of sodium amytal was injected in to the internal carotid artery, language was assessed using measures of counting, comprehension of commands, object naming, phrase repetition, sentence reading, and a rating of paraphasic errors during the period of hemianesthesia. Patients were subsequently shown six items for memory testing, which commenced when the EEG returned to baseline. Each patient was asked to recall a sentence and a picture, and then the six items on free recall. If all items were not recalled on free recall, each patient was shown a number of objects and was asked to identify the correct object. Two points were given for each correct answer on free recall and one point for each correct answer on recognition, resulting in a maximum possible score of 12 points. Five points or less was considered to indicate impairment. After a period of 30 min, the catheter was placed in the contralateral internal carotid artery and the procedure was repeated. A judgement regarding each patient's ability to co‐operate with the procedure was made. The side of HLD was determined by the patient's ability to understand and produce language on the above measures during hemianesthesia; language was considered to be unilateral following injection into a hemisphere where there was interrupted speech output and impaired comprehension with a significant failure to recall information for the tasks presented compared to injection into the contralateral hemisphere where speech production and comprehension were intact. Language was considered bilateral when there were no obvious differences in the performance following injection to either hemisphere. Recording of language function was performed by a clinical neuropsychologist with extensive experience of Wada testing (GB).

### 2.3 MRI acquisition

T1‐weighted images were acquired using a 1.5 T SIGNA whole body MR imaging system (GE Medical Systems, Milwaukee, WI) at the Magnetic Resonance and Imaging Analysis Research Centre (MARIARC), University of Liverpool. A spoiled gradient echo (SPGR) pulse sequence (TE = 9 ms, TR = 34 ms, flip angle = 30°) produced 124 coronal T1‐weighted images with a FOV of 20 cm. Each image refers to a contiguous section of tissue of 1.6 mm thickness, with in‐plane voxel size of 0.76 mm × 0.76 mm. Acquisition time was 13 min and 56 s for a 1 NEX scan.

### 2.4 MRI analysis

**Figure 1:**
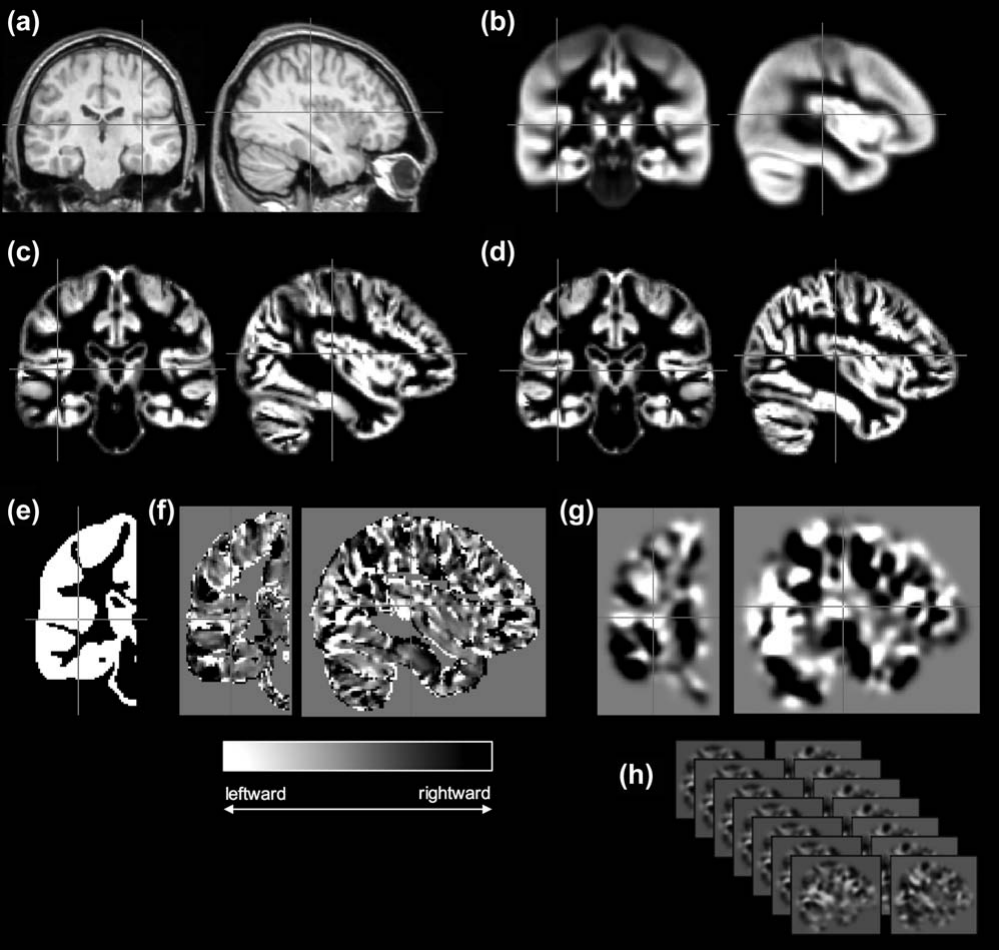
Overview of image processing for VBA. From acquired T1‐weighted images (a), symmetric templates are generated (b), which are used to obtain optimally segmented grey matter partitions (c), and corresponding flipped mirror image partitions (d). From these data, voxel‐wise AI images are generated (f), which are constrained to the right hemisphere using a right hemisphere binary mask (e). AI images are smoothed (g) and compared between groups (h) using conventional general linear models in SPM. Crosshairs indicate the left Heschl's gyrus throughout all stages of processing (other than in d, where the right Heschl's gyrus is indicated)

To map interhemispheric structural asymmetries over the whole brain, the VBA approach as described by Kurth et al. (2015) was applied. An overview of the main aspects of image processing for VBA is provided in Figure 1. VBA is based on many of the principles of conventional voxel‐based morphometry (Ashburner & Friston, 2000) with modifications to determine probabilistic volume asymmetries of homologous left‐right voxels across the brain. Initially, all images were processed using the VBM8 toolbox (http://dbm.neuro.uni-jena.de/vbm/) running in SPM8 (http://www.fil.ion.ucl.ac.uk/spm/software/spm8) and using DARTEL registration algorithms (Ashburner, 2007) to generate optimally spatially normalized grey and white matter compartments. Mirror image tissue‐specific segmentations of grey and matter were obtained for each patient by flipping with respect to the midline and a symmetric DARTEL template was generated, to which original and flipped tissue segments were warped, which included modulation using the registration flow fields. As all asymmetry index (AI) information is contained in one hemisphere (greater directional volume asymmetry of a given voxel to the left or right hemisphere) a right hemisphere mask was generated in symmetric template space to limit analysis to this hemisphere (Kurth et al., 2015). AI images were generated using an asymmetry formula for each homologous voxel using the original and flipped grey matter segments constrained to the right hemisphere:

$$AI=\frac{\left(left-right\right)}{0.5\times \left(left+right\right)}$$

All AI images were smoothed with an isotropic smoothing kernel of 8 mm. Conventional voxel‐based statistical analyses were performed in context of the general linear model in SPM8. Within‐group statistical analyses to identify significant interhemispheric asymmetries were performed between homologous left–right voxels in (a) the right‐handed controls, (b) right‐handed patients with left‐sided seizure focus and left HLD, and (c) all patients with left HLD regardless of side of seizure focus and handedness. Subsequently an analysis was performed to directly contrast voxel‐wise asymmetries between (a) controls and patients with left HLD in one model and (b) left TLE patients with left HLD, left TLE patients with right HLD, and left TLE patients with bilateral language representation in a second model. All results were reported using a correction for multiple comparisons at the cluster level (*p* < .05, FWE) (Kurth et al., 2015). In group comparisons, mean AI and hemispheric grey matter content were extracted for each significant cluster to determine the directionality of intergroup differences in asymmetries, which were imported into SPSS (v22.0; IBM SPSS Statistics, Armonk, NY) for analysis.

## 3 RESULTS

### 3.1 Wada test and clinical data

One hundred and fourteen (84.4%) patients had left HLD, 10 (7.4%) right HLD, and 11 (8.2%) bilateral language representation. Comparison of demographic and clinical data for these groups is provided in Table 1. Fifty‐six (49.1%) and 47 (41.2%) patients with left HLD had left and right onset epilepsy, respectively. This contrasted to 10 (100%) and zero in patients with right HLD, and nine (81.8%) and two (18.2%) patients with bilateral HLD. This represented a statistically significant difference in side of seizure onset between left and right HLD groups (χ^2^ = 7.81, *p* = .005), but not between left and bilateral HLD groups (χ^2^ = 2.04, *p* = .15). A multivariate ANOVA revealed a significant effect of group on handedness (*F* = 13.68, *p* < .001). Post‐hoc Bonferroni testing revealed that patients with bilateral (*p* = .05) and right (*p* < .001) HLD were significantly less right handed relative to patients with left HLD. There were no other significant differences between HLD groups.

**Table 1:** Breakdown of demographic and clinical information with respect to the three hemispheric language representation groups

	Age	F/M	Handedness	Age of onset	Duration of epilepsy	Side of epilepsy (L/R/B/U)
Left HLD	33 (9.5)	56/58	85.4 (40.6)	11.5 (8.7)	21.1 (11.1)	56/47/2/9
Right HLD	40 (9.6)	6/4	4.5** (93.7)	10.9 (9.0)	29.1 (9.4)	10/0/0/0
Bilateral HLD	33 (8.4)	6/5	46.3* (83.4)	8.2 (7.1)	24.7 (6.7)	9/2/0/0

*Note*. Abbreviations: B = bilateral; L = left; R = right; U = undetermined.

Age, handedness, age of onset, and duration of TLE values are mean (and *SD*). Male and female and side of TLE are number.

*Significantly different from left HLD (*p* = .05).

**Significantly different from left HLD (*p* < .001).

### 3.2 VBA: Within‐group cerebral asymmetries

**Figure 2:**
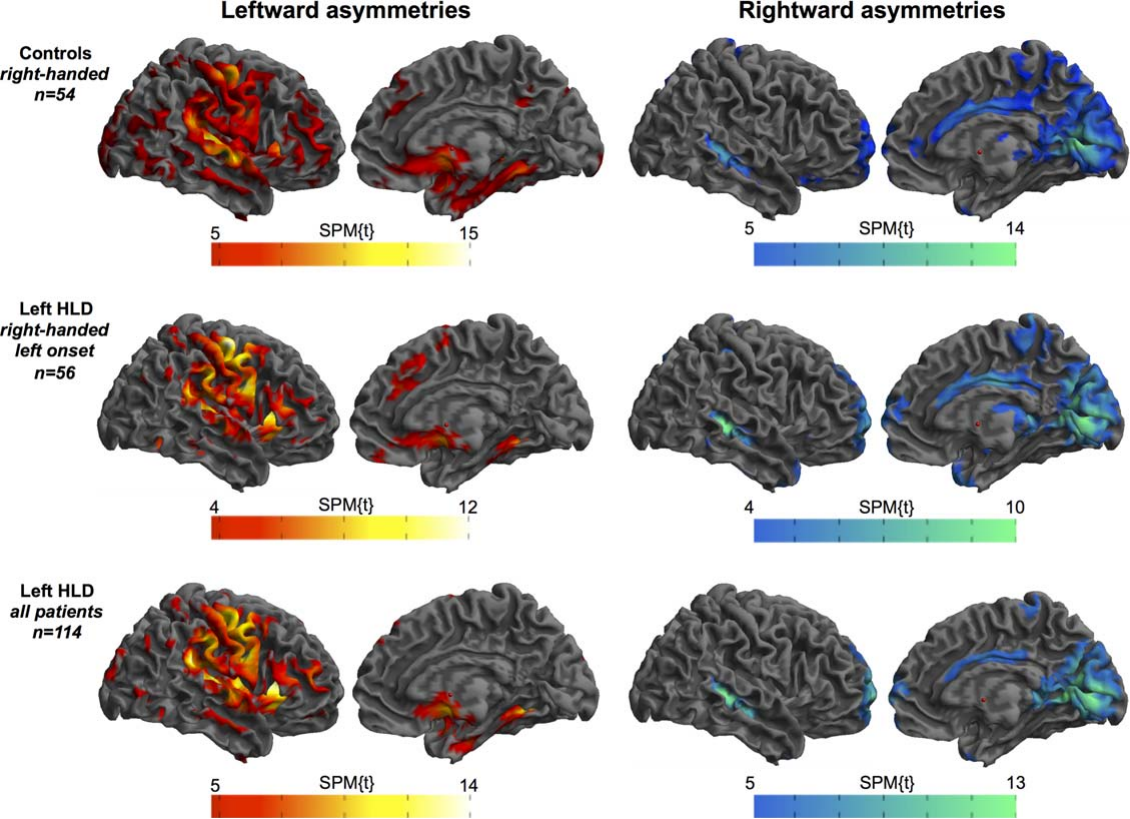
Significant leftward (red–yellow) and rightward (blue–green) interhemispheric asymmetries in controls and patients with left HLD [Color figure can be viewed at http://wileyonlinelibrary.com]

Figure 2 shows the significant leftward (yellow‐red) and rightward (green‐blue) interhemispheric asymmetries in (a) all controls, (b) all patients with left HLD, left TLE and right‐handedness (EHI = 50+, *n* = 56), and (c) all patients with left HLD, separately. The general topology of cortical asymmetries was relatively consistent across groups. Leftward asymmetries were observed in motor cortex, premotor cortex, inferior frontal gyrus, anterior and posterior insula, dorsal regions of the temporal lobe including the planum temporale and superior temporal gyrus, supramarginal gyrus, lateral and mesial temporo‐occipital cortex, mesial superior frontal gyrus, lateral cerebellum (not pictured), and accumbens area. Leftward asymmetry of anterior and middle portions of the mesial temporal lobe was only seen in controls. Rightward asymmetries were observed in the banks of the superior temporal and cingulate sulci, mesial occipital cortices, precuneus (notably less in the whole cohort of patients with left HLD), mesial cerebellum (not pictured), frontal pole, temporal pole, thalamus (predominantly the pulvinar region), and hippocampus (only in both left HLD patient groups).

In a supplementary analysis, a group‐wise comparison of asymmetries between the right‐handed controls and right‐handed patients with left HLD and left TLE (*n* = 56) was performed and the result is shown in Supporting Information, Figure 1. Only two clusters were identified as significantly different between groups, which corresponded to (a) the hippocampus (peak voxel 24 −34 −6 SPM_{t}_ = 7.84, *p*_FWE_ < .001) and (b) a smaller cluster in the superior temporal gyrus (peak voxel 63−28 4 SPM_{t}_ = 4.97, *p*_FWE_ = .02). These effects corresponded to significantly increased rightward asymmetry in patients relative to controls, which was due to greater volume loss of the left hippocampus (and left superior temporal gyrus) in patients.

### 3.3 VBA: Effects of HLD on cerebral asymmetries

Given that there was a significant effect of side of epilepsy on HLD, we restricted group comparison analyses of HLD in patients with the same diagnosis (i.e., unilateral left TLE). This resulted in a comparison between 56 patients with left HLD, 10 patients with right HLD, and nine patients with bilateral HLD (Table 1).

**Figure 3:**
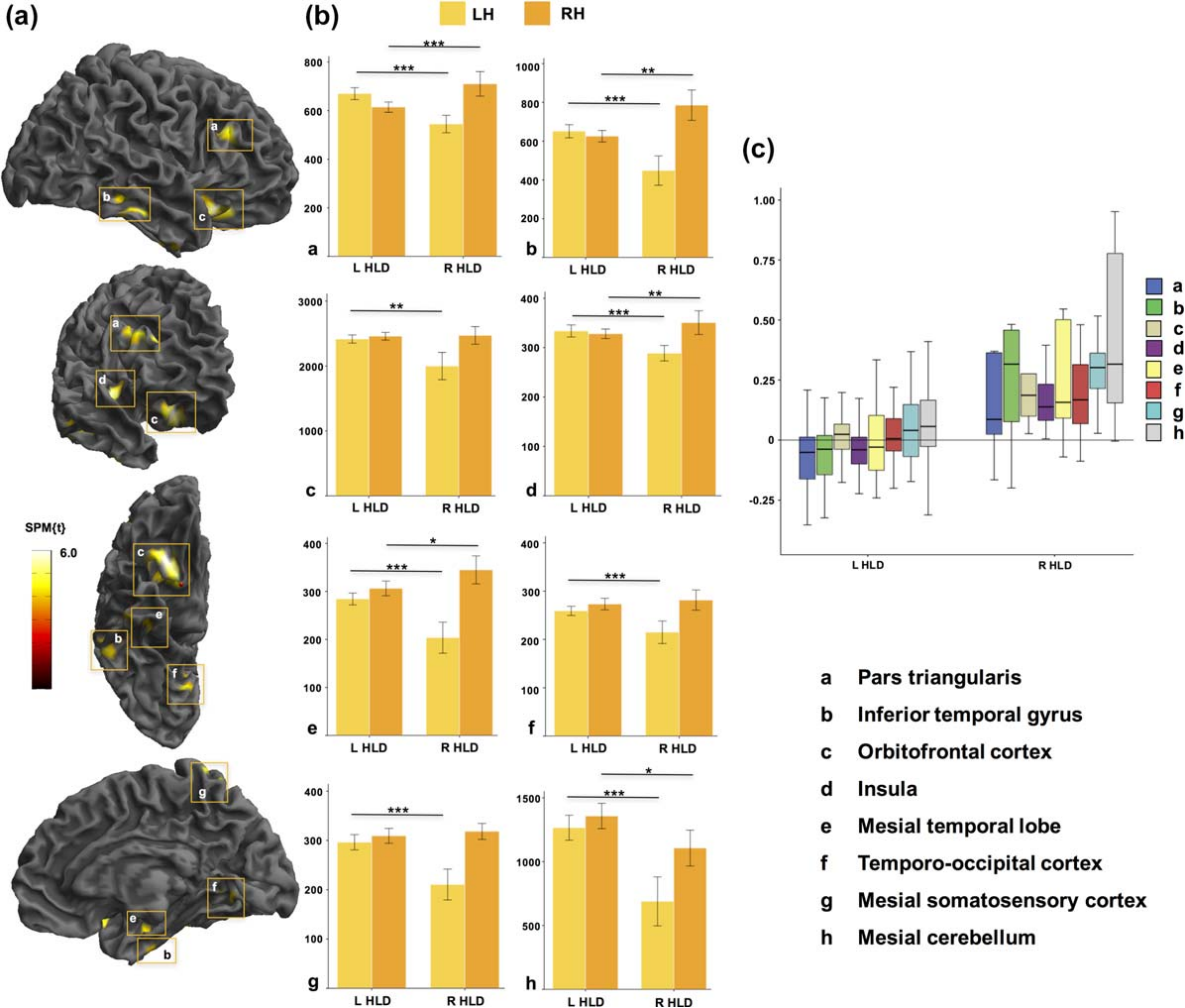
Significant differences in voxel‐based asymmetries between patients with left and right HLD: greater *rightward* asymmetries in right HLD relative to left HLD. (a) 3D rendered views of areas showing significantly different directional asymmetries between groups. (b) Histograms showing the amount of grey matter (number of voxels, *y*‐axis) in each cluster for the left and right hemisphere for both HLD groups. (c) Boxplots of interhemispheric asymmetries of clusters found to be significantly different. *y*‐axis corresponds to the asymmetry index. Letters correspond to the same regions highlighted in (a). Abbreviations: L, left; LH, left hemisphere; R, right; RH, right hemisphere. Mesial cerebellar clusters (h) are not shown on 3D renderings of cerebral hemispheres. ***p < .0001; **p = .001 to *p* = .01; *p < .05 [Color figure can be viewed at http://wileyonlinelibrary.com]

Results from comparisons between patients with left and right HLD indicated selective differences in the magnitude and/or direction of cerebral asymmetries in 11 specific brain regions across two contrasts (Table 2). The first contrast, corresponding to greater rightward asymmetry in patients with right HLD relative to those with left HLD, yielded eight statistically significant voxel clusters located in insula, orbitofrontal cortex, inferior temporal gyrus, dorsal area of pars triangularis of inferior frontal gyrus, mesial somatosensory cortex, mesial cerebellum, ventral temporo‐occipital cortex, and mesial temporal lobe (Figure 3). These asymmetries were found to be principally due to a relative reduction of grey matter in all these regions in the nondominant hemisphere, and additionally by a relative increase in the dominant hemisphere in insula, inferior temporal gyrus, mesial cerebellum, pars triangularis, ventral temporo‐occipital cortex, and mesial temporal lobe in patients with right HLD relative to left HLD (Figure 3b). Further examination of these findings revealed that mean group‐wise asymmetry of the insula, inferior temporal gyrus, pars triangularis, and mesial temporal lobe were leftward and orbitofrontal cortex, ventral temporo‐occipital cortex, somatosensory cortex, and mesial cerebellum slightly rightward, in patients with left HLD (Figure 3c). This contrasted to strong rightward asymmetry of all eight aforementioned regions in patients with right HLD (Figure 3c).

**Table 2:** VBA results for left HLD–right HLD comparisons

Contrast	Location	Peak coord	Peak *t*	Peak *p_FWE_*	*k*	*k*, *p_FWE_*	LH, *F*	LH, *p*	RH, *F*	RH, *p*
Right > left	Insula	39 −21 −2	6.24	0.001	155	0.02	3.86	0.052	11.19	0.001
	Orbitofrontal cortex	24 26 −20	6.19	0.001	1111	0.001	0.03	0.85	25.4	0.001
	Inferior temporal gyrus	52 −24 −20	6.14	0.006	354	0.001	19.76	0.0001	25.77	0.001
	Pars triangularis	43 32 22	6.04	0.004	388	0.001	14.51	0.0001	20.86	0.0001
	Mesial somatosensory cortex	9 −48 73	5.95	0.001	184	0.01	0.3	0.59	22.85	0.0001
	Cerebellum	20 −57 −50	5.78	0.001	829	0.001	5.15	0.03	25.92	0.0001
	Temporo‐occipital cortex	9 −60 −5	5.32	0.007	146	0.02	0.36	0.55	15.19	0.0001
	Mesial temporal lobe	38 −10 −29	4.44	0.16	171	0.02	4.76	0.03	28.95	0.0001
Left > right	Posterior precuneus	6 −58 33	6.45	0.001	152	0.02	0.63	0.8	23.23	0.0001
	Gyrus rectus	2 24 −26	5.56	0.003	114	0.04	6.94	0.01	17.75	0.0001
	Lateral temporo‐occipital junction	43 −57 16	4.75	0.05	117	0.04	10.54	0.002	2.67	0.11

*Note*. Abbreviations: *F* = ANOVA statistic; *k* = cluster size.

Peak *t* and *k* statistics are output from SPM. *F* statistics are output form SPSS based on extracted number of voxels in each cluster for the left and right hemisphere for each participant.

The reversed SPM contrast revealed that patients with right HLD had significantly increased leftward asymmetry of three clusters of voxels compared to patients with left HLD. These clusters were located in the lateral temporo‐occipital junction, gyrus rectus and posterior precuneus (Table 2 and Supporting Information, Figure 2). These differences were due to a combination of increasing volume in the nondominant, and decreasing volume in the dominant hemisphere in patients with right HLD relative to those with left HLD. Mean asymmetries of these three regions were in opposite directions; rightward in patients with left HLD and leftward in those with right HLD (Supporting Information, Figure 2B). There were no significant differences in cerebral asymmetries between patients with left HLD and bilateral language representation groups. Including age of onset and duration of epilepsy as confounding covariates in statistical models did not affect the pattern of results.

### 3.4 Left versus right HLD: Single‐subject findings

**Figure 4:**
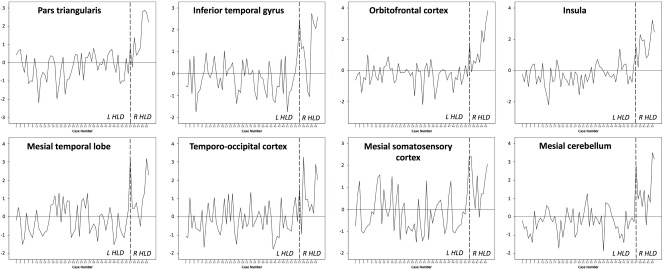
Individual patient plots of standardized AIs for the eight clusters found to be significantly different between patients with left and right HLD. The standardized mean AI is 0 (horizontal line). A negative or positive value indicates the patients that show an AI pattern resembling the group level pattern in the left (L) HLD or right (R) HLD group, respectively. Patients with a positive standardized AI in the left HLD group and those with a negative standardized AI in the right HLD group do not show consistency with the group level pattern for each region. The number and percentage of patients showing consistency is provided in Table 3. The broken vertical line indicates the division between patients with left and right HLD

We explored the proportion of individual patients resembling the group level findings in the left versus right HLD group comparisons by extracting grey matter AIs for each individual patient within the eight clusters found to be significantly different in group comparisons and converting individual AIs to z‐scores. These findings are provided in Table 3 and Figure 4. These results indicated that between 56.1% (pars triangularis) and 68.4% (inferior temporal gyrus; temporo‐occipital cortex) of patients with left HLD had standardized AIs that resembled the group pattern of asymmetry. Conversely, between 80% (mesial somatosensory cortex) and 100% (orbitofrontal cortex) of patients with right HLD resembled the group pattern of asymmetry. 90% of patients with right HLD showed this pattern for the remaining six brain regions. These results suggest more variability in interhemispheric asymmetry in patients with left HLD relative to those with right HLD.

**Table 3:** Number and percentage of patients’ standardized AIs consistent with the group level pattern for the eight clusters found to be significantly different between patients with left and right HLD (Figure 4)

		Pars triangularis	Inferior temporal gyrus	Orbitofrontal cortex	Insula	Mesial temporal lobe	Temporo‐occipital cortex	Mesial somatosensory cortex	Mesial cerebellum
Left HLD	*N*	32	39	37	36	34	39	34	37
	%	56.1	68.4	64.9	63.2	59.6	68.4	59.6	64.9
Right HLD	*N*	9	9	10	9	9	9	8	9
	%	90	90	100	90	90	90	80	90

## 4 DISCUSSION

In a large consecutive sample of Wada tested patients, we first sought to determine whole‐brain structural asymmetries in patients with left HLD and to compare these asymmetries with those observed in healthy controls. We found that controls, who were all right handed, and right‐handed patients with left HLD had comparable leftward asymmetries in motor regions, insula, dorsal temporal lobe areas, supramarginal gyrus, temporo‐occipital cortex, superior frontal gyrus, lateral cerebellum, and accumbens area, and rightward asymmetries in the banks of the superior temporal and cingulate sulci, mesial occipital cortices, precuneus, mesial cerebellum, frontal pole, temporal pole, and thalamus. In direct group comparisons, there was significantly greater rightward asymmetry of the mesial temporal lobe and a small region in the superior temporal gyrus in right‐handed patients with left HLD relative to controls, which were likely due to expected volume loss of these left temporal lobe structures in patients. Second, we directly compared structural asymmetries between patients with left HLD with those with right and bilateral language representation, all of whom had a left‐sided temporal seizure focus, to determine the neuroanatomical correlates of language dominance. We found that patients with left and right HLD had significant differences in asymmetry of insula, pars triangularis, inferior temporal gyrus, orbitofrontal cortex, ventral temporo‐occipital cortex, mesial temporal lobe, and cerebellum. These asymmetries were largely influenced by a decrease in volume of homologous cortex in the nondominant hemisphere, and an increased volume in the dominant hemisphere, in patients with right HLD relative to those with left HLD. There was also a difference in handedness between patient HLD groups; those with right HLD and bilateral language representation were significantly less right handed than patients with left HLD. We discuss the biological interpretations of these findings before highlighting pertinent methodological considerations.

### 4.1 Biological implications

Interhemispheric asymmetries of frontal and occipital cortices, insula, planum temporale, superior temporal sulcus, cingulate sulcus, temporal pole, thalamus, and cerebellum, which we observed in healthy right‐handed controls and patients with left HLD, have been reported in previous VBA studies in healthy controls (Good et al., 2001; Takao et al., 2011; Watkins et al., 2001). Furthermore, leftward supramarginal gyrus asymmetry is consistent with a previous study that examined surface area asymmetries (Lyttelton et al., 2009). Asymmetries of anterior speech region, which were also observed in controls and patients, are much less consistently observed (see below). Importantly, directional asymmetries were consistent between patients with the most common functional brain organization (left HLD and right handedness) and right‐handed controls, apart from asymmetry of the hippocampus and a circumscribed region of the superior temporal gyrus. This suggests that structural asymmetry–HLD relationships observed in patients likely exist in the healthy brain, with potentially the exception of mesial temporal lobe in particular, which is the primary pathological brain region in patients with TLE (Thom, 2014). However, mesial temporal asymmetries were associated with HLD in patient group comparison analyses matched for side of TLE. Previous work has suggested the importance of the hippocampus for HLD; Liegeois et al. (2004) demonstrated right HLD in children with early lesions to left hippocampus, and Jansen et al. (2010) reported increased right hippocampal grey matter density in healthy people with right HLD relative to those with left HLD using voxel‐based structural methods. Therefore, the importance of the mesial temporal lobe for HLD should not be overlooked.

We report that HLD is related to directional asymmetries of the insula. Based on manual stereological measurements, we were the first to report asymmetry–HLD relationships of the insula in healthy people (Keller et al., 2011). The potential existence of this relationship was previously hypothesized (Flynn, Benson, & Ardila, 1999), based on the insula's role in language articulation from functional neuroimaging studies (Blank, Scott, Murphy, Warburton, & Wise, 2002; Price, 2000, 2010; Wise, Greene, Buchel, & Scott, 1999), damage to the insula in patients with aphasia due to cerebral infarction (Mohr, 1979; Mohr et al., 1978), and historical anatomical reports documenting morphological asymmetries of the insula (Clark, 1896; Cunningham, 1891, 1892). There is now a growing literature further documenting correlation between structural asymmetries of the insula and HLD in healthy people (Bidula & Kroliczak, 2015; Chiarello et al., 2013; Greve et al., 2013; Keller et al., 2011). Population‐wise leftward asymmetry of insula has been shown in people with undetermined HLD (Van Essen, Glasser, Dierker, Harwell, & Coalson, 2012; Watkins et al., 2001). We had initially reported differences in mean directional asymmetry of the insula in people with left and right HLD (Keller et al., 2011), such that left HLD was associated with leftward insular asymmetry and right HLD was associated with rightward insular asymmetry. However, reduction in leftward asymmetry in people with right HLD, and not necessarily a shift toward rightward asymmetry, has been reported in several studies (Bidula & Kroliczak, 2015; Chiarello et al., 2013; Greve et al., 2013). Results from this study indicate a group‐wise leftward asymmetry of insula in people with left HLD and rightward asymmetry in people with right HLD (Figure 3), which further supports our original observations (Keller et al., 2011). The difference in directional asymmetry between HLD groups appears to be due to a relative reduction of insular grey matter in the left insula and an increase in the right insula in patients with right HLD relative to those with left HLD. As cited in our previous study (Keller et al., 2011), Cunningham (1892) wrote “… it (is) probable that at all periods of life there is a relatively greater development of the island of Reil [insula] on the left side than on the right. The difference in extent of the insula on the two sides of the brain is very slight, but it is seen in every stage (of development)” (p. 109). In light of results presented here, it appears possible that this development is attenuated in people with right HLD, and potentially that compensatory mechanisms drive increasing right‐sided volume in these patients. Whether these alterations are an abortive (i.e., due to acquired injury) or natural feature of neurodevelopment is an unresolved issue. Right HLD in people with no known neurological injury has not been associated with intellectual or cognitive impairment in large pragmatic studies (Bishop, 2013; Knecht et al., 2001) or recruitment of atypical language functional cortical networks (Knecht et al., 2003).

The insula is cytoarchitectonically divided into three main regions (although 7–8 smaller subdivisions can be identified (Gallay, Gallay, Jeanmonod, Rouiller, & Morel, 2012; Morel, Gallay, Baechler, Wyss, & Gallay, 2013)) including anterior agranular region (Ia) that has a role in olfactory and autonomic functions, the middle dysgranular region (Id) that has a role in gustatory functions, and posterior granular region (Ig) that is associated with somatosensory, auditory and visual functions (Chikama, McFarland, Amaral, & Haber, 1997; Flynn et al., 1999; Mesulam & Mufson, 1982a, 1982b). The control of several of these insular functions is essential for the smooth, coordinated production of speech (Craig, 2003, 2009). The anterior‐to‐posterior agranular‐to‐granular organization of the insula reported in previous anatomical studies of the macaque monkey has been putatively replicated in an in vivo neuroimaging study in humans (Cerliani et al., 2012). There is evidence from meta‐analysis fMRI studies indicating that the insula has importance for both the motoric aspects of speech production and receptive language functions (Ardila, Bernal, & Rosselli, 2014; Oh, Duerden, & Pang, 2014), and that the insula may play a coordinating role in interconnecting anterior (speech) and posterior (receptive) language systems (Ardila, Bernal, & Rosselli, 2016). However, there is a paucity of studies that have directly addressed the functional neuroanatomy of the insula with respect to receptive language functions. Furthermore, to our knowledge, all HLD–cerebral asymmetry studies published to date that have used functional imaging and the Wada test have been predominantly assessments of speech production; HLD in these cases (including in this study) should more realistically be termed hemispheric speech dominance.

The association between insula and orbitofrontal cortex is interesting. We report concomitant directional asymmetries of insula and orbitofrontal regions that are consistent with the side of HLD. Some work failed to find clear cytoarchitectonic borders between anterior insula and posterior orbitofrontal cortex (Mesulam & Mufson, 1982a), whereas others report that the anterior agranular insula extends onto the posterior orbitofrontal cortex (Carmichael & Price, 1994). The cortical connectivity profiles of the insula and lateral orbitofrontal cortex are similar; “These common connectivity patterns support the conclusion, based on architectonic observations, that the insulo‐orbito‐temporopolar component of the paralimbic brain should be considered as an integrated unit of cerebral organization” (Mesulam & Mufson, 1982b; p. 38). Therefore, our findings indicating associations between insula, orbitofrontal cortex, and temporal regions and HLD may reflect an underlying anatomical network that supports the hemispheric lateralization of language functions.

Similar to the pattern of asymmetries observed for the insula, there was a general leftward structural asymmetry of the pars triangularis in patients with left HLD and rightward asymmetry in those with right HLD. There are inconsistencies in the literature with respect to cerebral asymmetries of the anterior speech region and HLD. In Wada tested patients, both Foundas et al. (1996) and Dorsaint‐Pierre et al. (2006) report such relationships (pars triangularis and pars opercularis, respectively). In healthy people, Josse et al. (2009) report voxel‐based correlations between structural asymmetries of the anterior speech region and functional asymmetries during language functional MRI tasks. However, these relationships have not been observed in other studies of healthy people (Chiarello et al., 2013; Greve et al., 2013; Jansen et al., 2010; Keller et al., 2011). In a review article, we previously suggested that this lack of consistency might be due to different image analysis methods having been used especially given that, at that time, most ROI approaches did not identify structural asymmetry of Broca's area, whereas automated whole‐brain approaches were more likely to (Keller et al., 2009a). However, in light of more recent studies (Bidula & Kroliczak, 2015; Chiarello et al., 2013; Greve et al., 2013; Jansen et al., 2010; Josse et al., 2009; Keller et al., 2011), a relationship between image analysis approach and Broca's area asymmetry findings is not obvious. For example, voxel‐based (Dorsaint‐Pierre et al., 2006; Jansen et al., 2010; Josse et al., 2009) and manual delineation (Foundas et al., 1996; Keller et al., 2011) approaches do not report consistent findings on the relationship between structural asymmetry of Broca's area and HLD. Interestingly, studies using Freesurfer vertex‐wise methods in healthy people tend not to demonstrate a relationship between HLD (or putative measures of HLD) and structural asymmetry of the constituent regions of Broca's area (Bidula & Kroliczak, 2015; Chiarello et al., 2013; Greve et al., 2013).

Our findings concerning the relationship between structural asymmetries and HLD were obtained in comparisons between left and right HLD. Patients with bilateral language representation and left HLD, matched for left TLE, did not significantly differ in terms of cerebral asymmetries. Given that no differences were observed between patients with left HLD and those with bilateral language representation, we may conclude that cerebral asymmetries of cortical language regions appear to be increasingly similar in people with left and bilateral HLD relative to people with right HLD. This may suggest that right HLD is mediated by different neurodevelopmental mechanisms compared to left and bilateral HLD, and categorizing bilateral and right HLD together as “atypical” HLD may be inappropriate.

### 4.2 Methodological considerations

Pathological alterations in brain structure in patient samples (e.g., grey matter atrophy) may call into question whether asymmetries observed in patients would also be observed in the healthy brain. However, asymmetry–HLD relationships observed in patients with epilepsy are also seen in healthy people (Dorsaint‐Pierre et al., 2006). For example, the relationship between insular asymmetry and HLD seen in this study has been reported in healthy people (Bidula & Kroliczak, 2015; Chiarello et al., 2013; Greve et al., 2013; Keller et al., 2011), suggesting this is a natural structure–function relationship rather than one that is caused by injury or disease. Furthermore, unlike previous studies of cerebral asymmetry in Wada tested patients (Dorsaint‐Pierre et al., 2006; Ellmore et al., 2010; Foundas et al., 1994, 1996; Matsumoto et al., 2008), we were able to compare asymmetries between patients matched in terms of location and type of epilepsy, given the large number of consecutively assessed patients included in this study. In particular, significant differences in structural asymmetries shown in Figure 3 were obtained from VBA comparisons between patients with left and right HLD who were all diagnosed with left TLE and had comparable age of onset of epilepsy. As expected, patients with right HLD had significantly reduced incidence of right‐handedness, which is a reproducible finding (Knecht et al., 2000b; Pujol et al., 1999). We did not use handedness as a confounding covariate in our statistical models as this would interact with the HLD–asymmetry differences, given the interaction between HLD and handedness. Our aim was not to disentangle the relationships between HLD and handedness, but to identify the interhemispheric anatomical correlates of HLD in Wada tested patients whilst controlling for potentially confounding clinical aspects of epilepsy, such as side of seizure onset.

VBA, like conventional voxel‐based morphometry, has advantages and disadvantages. The primary advantage is that asymmetry analyses are not restricted to any particular grey matter region, including those areas most commonly associated with HLD (i.e., Broca's area, planum temporal, and insula). Without a whole‐brain approach, we would not have identified relationships between HLD and asymmetry of other regions, such as orbitofrontal cortex and tempro‐occipital cortex. However, we have not analyzed white matter asymmetries, which is better studied using DTI than voxel‐based analysis of T1‐weighted images (Keller & Roberts, 2008). Thus there remains the possibility of a relationship between white matter architectural asymmetry and HLD, despite inconsistencies reported in previous DTI tractography studies (James et al., 2015; Powell et al., 2006; Vernooij et al., 2007). Notably, a previous study performed VBA on T1‐weighted and fractional anisotropy maps reconstructed from DTI data in healthy young people without known HLD and reported concomitant leftward asymmetry of the arcuate fasciculus and planum temporale (Takao et al., 2011). We did not acquire DTI data in our sample and therefore we were unable to determine the relationships between white matter asymmetry and HLD. Furthermore, although frequently employed, voxel‐based approaches are largely unable to determine the likely type of architectural alterations underlying group‐based differences. Surface‐based approaches, which can provide information on the surface area, curvature and thickness of cortex, offer an alternative method of investigating brain architecture that may provide more specific interpretations of structural–functional correlates (Van Essen, Drury, Joshi, & Miller, 1998; Winkler et al., 2010). Nevertheless, our voxel‐based findings do show consistencies with some surface‐based findings on the relationship between HLD and brain asymmetry in healthy people, particularly the insula (Bidula & Kroliczak, 2015; Chiarello et al., 2013; Greve et al., 2013). Finally, further work is required to progress our group‐level findings to inform subject‐specific classifications using, for example, linear support vector machine approaches (Cui, Xia, Su, Shu, & Gong, 2016).

The Wada test is invasive, costly, carries associated risk, and is only applicable to patients who are undergoing comprehensive evaluation for future neurosurgery (Beimer, Buchtel, & Glynn, 2015; Kemp et al., 2008; Loddenkemper, Morris, & Moddel, 2008). As such, there has been a significant endeavor to replace Wada testing with noninvasive functional neuroimaging techniques, including functional MRI, magnetoencephalography and functional transcranial Doppler sonography, and transcranial magnetic stimulation (Abou‐Khalil, 2007; Baxendale, 2009; Binder et al., 1996; Doss, Zhang, Risse, & Dickens, 2009; Knake et al., 2003; Knecht et al., 1998; Rihs, Sturzenegger, Gutbrod, Schroth, & Mattle, 1999; Theodore, 2003). In a meta‐analysis of Wada test and functional MRI concordance studies, HLD agreement between modalities was reported for 94% patients with left HLD and 51% patients with atypical HLD, leading the authors to conclude that fMRI should be used as a triage assessment and that Wada testing should be utilized when fMRI does not establish clear left HLD (Bauer, Reitsma, Houweling, Ferrier, & Ramsey, 2014). Other work has reported lower concordance between the Wada test and fMRI in patients with atypical HLD (Janecek et al., 2013). Although test–retest applications of the Wada test are rare, Loddenkemper, Morris, Lineweaver, and Kellinghaus (2007) reviewed results from 1,249 tests on 1,190 patients, and reported that HLD was reproduced in all but one of the 4% of patients in their sample who had the Wada test repeated. This level of HLD reproducibility far exceeds that of functional MRI, which carries a degree of subjectivity in the calculation of functional asymmetry indices (Fernandez et al., 2003; Rutten et al., 2002). The Wada test remains the only definitive method for identification of HLD (Ellmore et al., 2010) and represents a significant methodological strength of this study.

## 5 CONCLUSIONS

Using VBA, we have reported that healthy right‐handed controls and Wada tested right‐handed patients with left HLD have comparable volume asymmetries of brain regions previously reported to show structural asymmetries, with the exception of the brain region known to be pathological in patients (i.e., the hippocampus). In direct comparisons between patient groups based on the side of HLD, we report that HLD is related to interhemispheric asymmetries of a network of brain regions including insula, pars triangularis, inferior temporal gyrus, ventral temporo‐occiptal cortex, orbitofrontal cortex, and cerebellum. In people with right HLD, an alteration in the magnitude of directional asymmetries in these regions can be observed, which are due to a combination of a relative increase of grey matter in the dominant hemisphere and a reduction in the nondominant hemisphere. The anatomical basis of language lateralization in people with right HLD may be mediated by different mechanisms compared to both left HLD and bilateral language representation.

## CONFLICT OF INTEREST

The authors declare no competing financial interests.

## Supporting information

Additional Supporting Information may be found online in the supporting information tab for this article.

Supporting Information Figure 1

Supporting Information Figure 2
