# Supplementary material for: A voxel‐based asymmetry study of the relationship between hemispheric asymmetry and language dominance in Wada tested patients
Source: Hum Brain Mapp. 2018 Mar 23;39(7):3032–45. doi: 10.1002/hbm.24058 (PMC6055618; doi:10.1002/hbm.24058)
Supplement: Supplementary file 1 — Supporting Information Figure 1 [file HBM-39-3032-s001.docx]

**A voxel-based asymmetry study of the relationship between hemispheric asymmetry and language dominance in**

**Wada tested patients**

Supplementary Materials: Figure 1


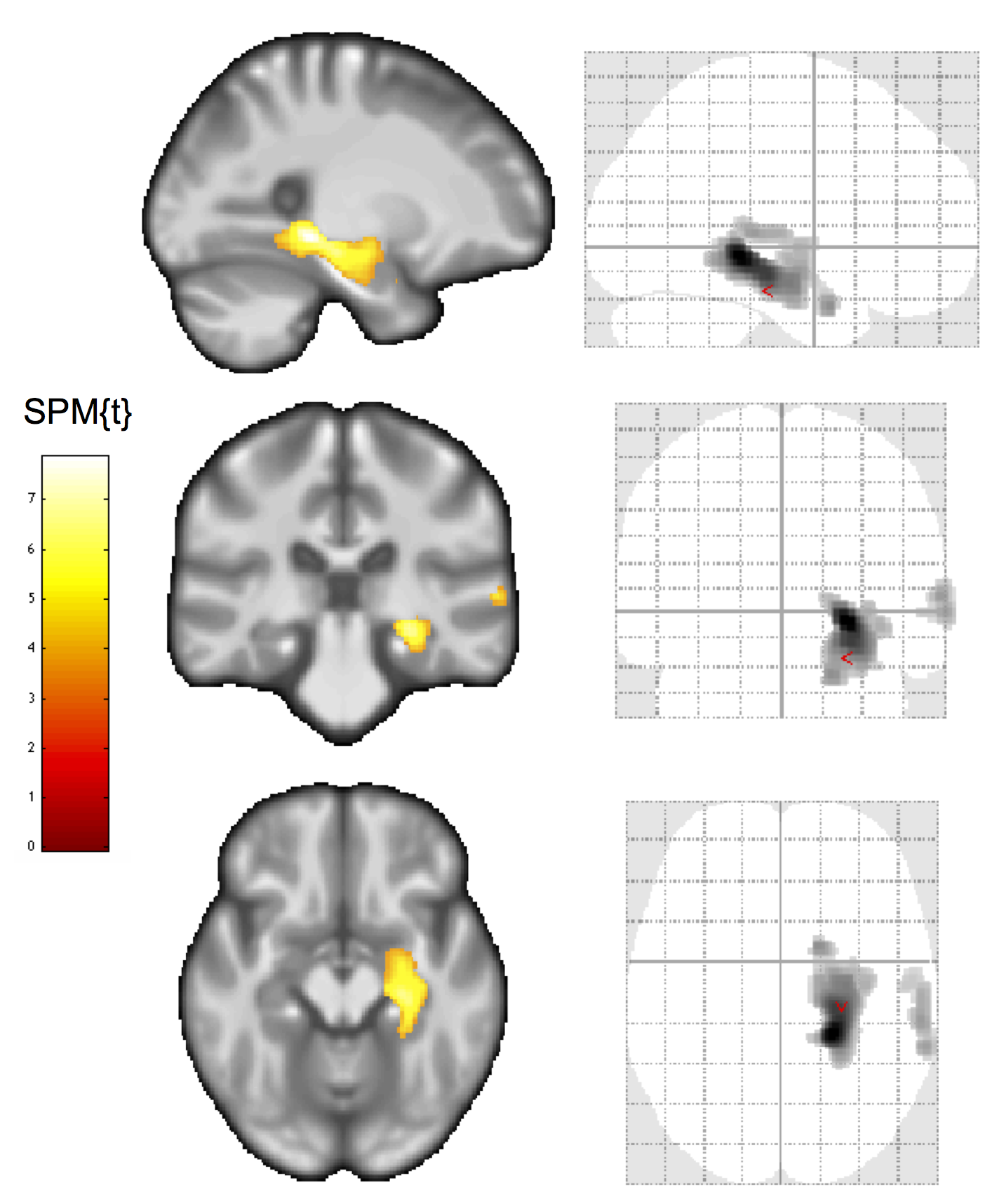


**Supplementary Figure 1**

Significant differences in brain asymmetries between controls and right-handed patients with left TLE and left HLD. Results are shown projected onto a template image (left) and using glass-brain views (right). Clusters represent significantly increased rightward asymmetry in patients relative to controls due to greater volume loss of these structures in the left hemisphere in patients.
