# Supplementary material for: A voxel‐based asymmetry study of the relationship between hemispheric asymmetry and language dominance in Wada tested patients
Source: Hum Brain Mapp. 2018 Mar 23;39(7):3032–45. doi: 10.1002/hbm.24058 (PMC6055618; doi:10.1002/hbm.24058)
Supplement: Supplementary file 2 — Supporting Information Figure 2 [file HBM-39-3032-s002.docx]

**A voxel-based asymmetry study of the relationship between hemispheric asymmetry and language dominance in**

**Wada tested patients**

Supplementary Materials: Figure 2


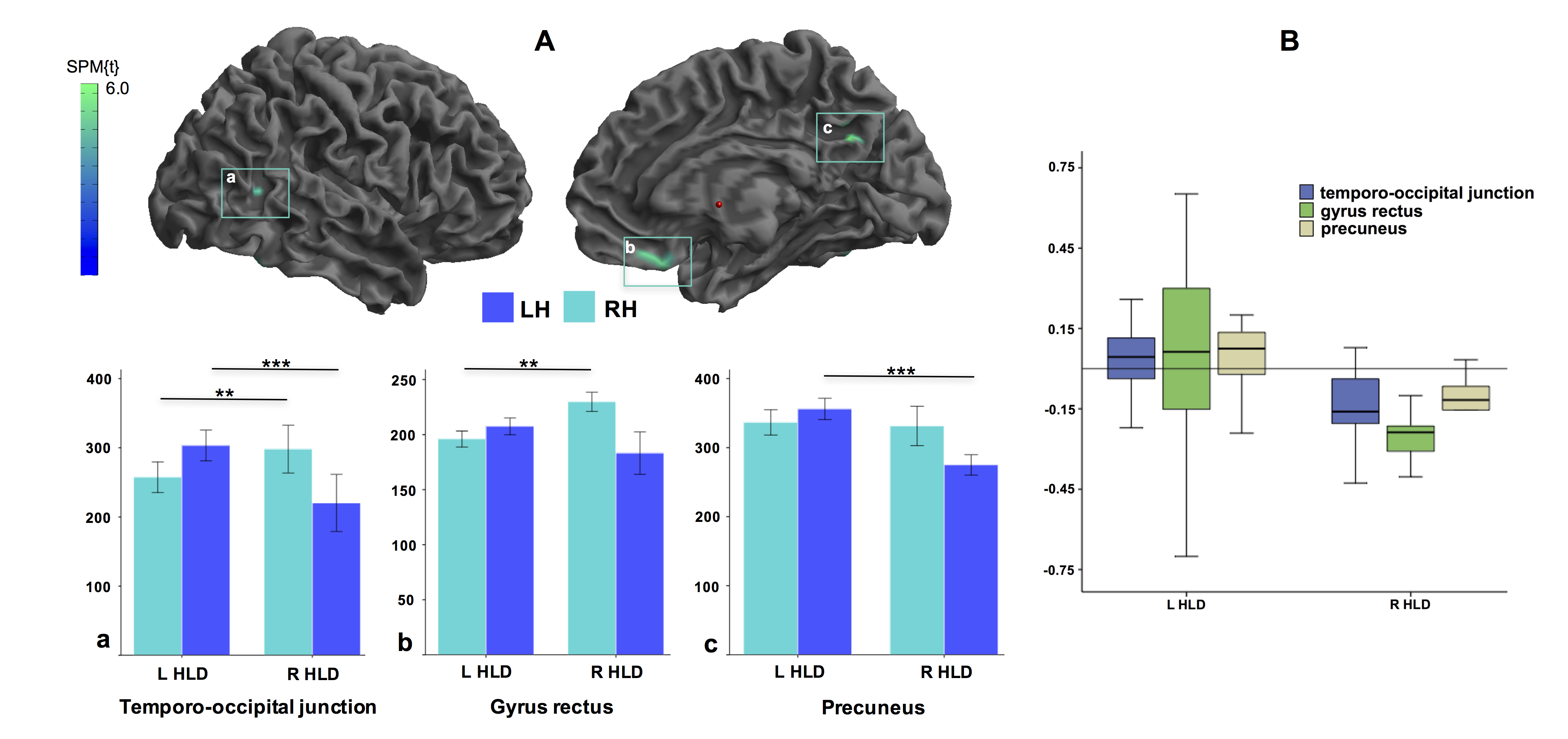


**Supplementary Figure 2**

Significant differences in voxel-based asymmetries between patients with left and right HLD: greater *leftward* asymmetries in right HLD. **A.** Top: 3D rendered views of areas showing significantly different directional asymmetries between groups. Bottom: histograms showing the amount of grey matter (number of voxels, *y*-axis) in each cluster for the left and right hemisphere for both HLD groups. Abbreviations: L, left; LH, left hemisphere; R, right; RH, right hemisphere. *** p<0.0001; ** p=0.001 – p=0.01. **B.** Boxplots of interhemispheric asymmetries of clusters found to be significantly more *leftward* in right HLD relative to left HLD. Letters correspond to the same regions highlighted in Figure 6. *y*-axis corresponds to the asymmetry index.
